# VegAnn, Vegetation Annotation of multi-crop RGB images acquired under diverse conditions for segmentation

**DOI:** 10.1038/s41597-023-02098-y

**Published:** 2023-05-19

**Authors:** Simon Madec, Kamran Irfan, Kaaviya Velumani, Frederic Baret, Etienne David, Gaetan Daubige, Lucas Bernigaud Samatan, Mario Serouart, Daniel Smith, Chrisbin James, Fernando Camacho, Wei Guo, Benoit De Solan, Scott C. Chapman, Marie Weiss

**Affiliations:** 1 UMR TETIS, CIRAD, Montpellier, France; 2 INRAE, Avignon Université, UMR EMMAH 1114, 84000 Avignon, France; 3 Arvalis, 228, route de l’Aérodrome - CS 40509, 84914, Avignon, Cedex 9 France; 4 HIPHEN SAS, 120 Rue Jean Dausset, Agroparc-Batiment Technicité, 84140 Avignon, France; 5 The University of Queensland, School of Agriculture and Food Sciences, Gatton, QLD 4343 Australia; 6 EOLAB, c/Savina,8.A4, 46980 Valencia, Spain; 7 Graduate School of Agricultural and Life Sciences, The University of Tokyo, Tokyo, 188-0002 Japan

**Keywords:** Computer science, Plant breeding

## Abstract

Applying deep learning to images of cropping systems provides new knowledge and insights in research and commercial applications. Semantic segmentation or pixel-wise classification, of RGB images acquired at the ground level, into vegetation and background is a critical step in the estimation of several canopy traits. Current state of the art methodologies based on convolutional neural networks (CNNs) are trained on datasets acquired under controlled or indoor environments. These models are unable to generalize to real-world images and hence need to be fine-tuned using new labelled datasets. This motivated the creation of the VegAnn - **Veg**etation **Ann**otation - dataset, a collection of 3775 multi-crop RGB images acquired for different phenological stages using different systems and platforms in diverse illumination conditions. We anticipate that VegAnn will help improving segmentation algorithm performances, facilitate benchmarking and promote large-scale crop vegetation segmentation research.

## Background & Summary

Over the last 10 to 15 years, there has been a growing interest in image-based plant studies using automated digital cameras. Computer vision is being widely adopted to access crop knowledge from these images for various applications including decision support in farms for irrigation or fertilization application, harvest planning, disease, weed management, crop identification and the computation of biophysical variables^1–5^. In the last decade, the availability of crop genomic information has accelerated numerous breeding programs^6,7^ and there is increasing use of extensive phenotypic measurements through the crop growth cycle to interpret the behavior of cultivars at finer time scales and to link the phenotype to the genotype^8^. Further, interpretation from image analysis, especially green fractional cover and leaf area index, can also be used for the validation and calibration of remote sensing products^9–11^.

These different applications are supported by the rapid development of robotic technologies associated with image acquisition and analysis workflows. For such standardized fully automated processing, RGB images are preferred as being low-cost, versatile and of high-spatial resolution. Crop traits of interest (e.g. green cover fraction, leaf area index, leaf spot disease, etc.) are often extracted from these images using fully-automated pipelines wherein semantic segmentation is performed as a critical intermediate step. This step, applied before other processing steps, is a pixel-level classification that isolates the vegetation from the background i.e. soil, rock, dead leaves, etc. Hereon referred to as “Vegetation Segmentation”, this is indeed a well-established area of research, with well-known drawbacks^12,13^.

Vegetation segmentation approaches can be described as being in three broad categories:

- **Color-based approaches**: Include thresholding applied on pixel color values, color-based indices such as excess green (ExG), vegetation index (VI) among others^14^. In most cases, such approaches require a user-defined threshold which often comes with a significant risk of dataset bias and lacks robustness and consistency across different datasets.
- **Machine learning approaches based on pixel-level features**: These approaches utilize features computed from the spectral information contained in the pixels and may also include the features computed from the different color-space representations. However, such colour-based techniques struggle to generalize over varying illumination conditions, chromatic aberrations which might cause some of the soil pixels to appear green and the quality of the camera optics. Further, in image regions saturated either by strong specular reflection or under-exposure, it is difficult to reliably classify the pixels only using the color information. Also, the pixel color might be misleading in certain situation. For example, soil appearing greenish due to the presence of algae or vegetation appearing brownish-yellow due to senescence. Additionally, the soil and crop residues in the background are difficult to distinguish from the senescent vegetation observed on the canopy since they encompass a similar range of brownish colors. Therefore, textural and contextual information should be exploited to overcome the aforementioned problems and better segment RGB images into vegetation and background.
- **Machine learning approaches based on color-texture-shape characteristics**: The methodologies within this category utilize the context and spatial information, in addition to the pixel values extracted from the images. To overcome the limitation of pixel-level features, researchers began using handcrafted features such as Bag of Words, SIFT, GLCM, Canny Edge Detectors, etc.^15,16^. Due to the high dimensionality of these features, a sizable amount of data is required to train the algorithms to distinguish between vegetation and background. Recent advances in deep learning methodologies have enabled automatic learning of the necessary features from the dataset, surpassing traditional hand-crafted features and machine learning approaches^17^.

Deep learning methodologies have achieved notable success for certain agricultural and phenotyping tasks especially to characterise crop ‘traits’, e.g.^18–22^. The application of these labelling for vegetation segmentation have therefore received increasing attention in the recent years^5,17^. The organization of challenges, conferences^23^ and availability of open labelled datasets under controlled conditions^17,24^ have eased the adoption of deep learning methods for vegetation segmentation. However, the public datasets are limited to specific illumination conditions, crop varieties and soil types. Deep learning models trained on such small, domain-specific datasets tend to perform poorly on new domains. Thus, a key reason for lack of deep learning solutions for real-world conditions is the lack of diverse, publicly available labelled dataset for vegetation segmentation cf other types of datasets like boundary box classifications^25–27^. The curation of a large pixel-level labelled dataset for vegetation segmentation is indeed an expensive and tedious task that requires contribution from experts.

This need motivated our creation of the VegAnn for outdoor vegetation segmentation from RGB images. To our knowledge, this is the first multi-crop image dataset for semantic segmentation that has been specifically constituted by sampling a large range of crop species, grown under diverse climatic and soil conditions. VegAnn assembles a total of 3775 images from various datasets with samples acquired over a large diversity of growing scenarios and throughout the crop growth cycle. This paper describes the dataset characteristics and shows how it can be used to develop a powerful crop segmentation algorithm. We also highlight the interest of merging datasets from different crop/species and provide baseline state of the art results on the VegAnn dataset^28^. We believe that this database will serve as a reliable tool for benchmarking new algorithms and eventually boost research on vegetation segmentation.

## Methods

### Annotation rules

**Fig. 1:**
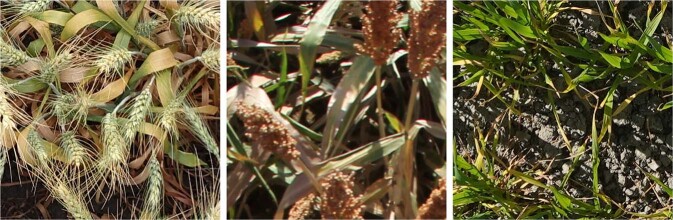
Example of images where the visual distinction of green vegetation parts from the senescent plant parts is not straightforward: leaves have roughly the same shape and texture but different colors.

VegAnn^28^ was annotated following a simple rule: all the pixels belonging to plants were labelled as vegetation (including stem, flowers, spikes, leaves - either healthy or senescent) and the rest as background (which includes crop residues or dead leaves present on the ground). This reduced potential bias among annotators since, for instance, excluding plant senescent leaves from the vegetation class would be prone to subjectivity. Indeed, the decision whether the vegetation is healthy or not is not straightforward as illustrated in the examples shown in Fig. 1.

Moreover, including the senescent part of the leaves within the vegetation class allows retention of information about leaf shape. This aligns with the reasoning of convolution-based approaches, since, in contrast to pixel-based methods, they utilize both the texture and the contextual information for decision making. Finally, it can be noticed that once the vegetation is extracted from the image, it is then relatively easy to use color-based methods to extract the non healthy parts that can no longer be confused with the background^29^.

**Fig. 2:**
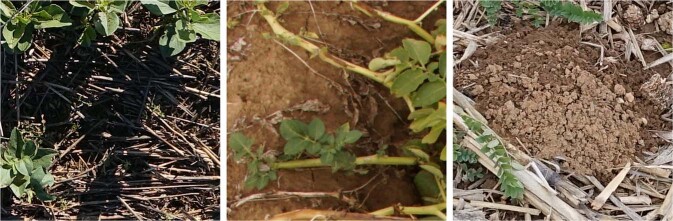
Example of images where crop residues are present at the ground level and considered as background in VegAnn.

Despite this simple annotation rule, there were cases where decision making was not unequivocal. For instance, with images containing crop residues as seen in Fig.  2. We therefore added a second rule notifying that dead plants present at the ground level are considered as background. The presence of residues is often observed when crop rotation is practiced. This kind of crop management has a good impact on carbon sequestration and is prevalent in many cropping systems.

### Creating VegAnn by assembling various sub-datasets of RGB images

The VegAnn dataset was aggregated from different sub-datasets collected by different institutions within the scope of various projects under specific acquisition configurations. This aggregation process encompassed a wide range of measurement conditions, crop species and phenological stages. The images were thus acquired using different cameras equipped with different focal length optics, at variable distances from the top of the canopy. An important requirement for the integration of external sub-dataset within VegAnn is to have downward-looking images that offer sufficient detail (i.e. spatial resolution) for accurate visual distinction between the vegetation and the background. The cameras were positioned at a few meters above the canopy with a ground sample distance (GSD) varying from 0.1 to 2 mm/ pixel. The original raw images (referred to as images in the following) were cropped into several patches of 512 × 512 pixels. The VegAnn dataset content was optimized by selecting images within all the sub-datasets so that they represent well the diversity of the samples while keeping a good balance between plant species, development stages, environmental and acquisition conditions.

**Table 1:** Datasets used to compose VegAnn.

Dataset name	Owner/Institute	Nb of Images	References
Literal	Arvalis	938	Na
Phenomobile	Arvalis, INRAE	225	Na
INVITA	University of Queensland	500	^33^
EasyPCC	The University of Tokyo	539	^38^
DHP	INRAE, EOLAB, NEON	948	^34^
P2S2	INRAE	499	^11^
Crowdsourcing	Open sources	82	Na

**Table 2:** VegAnn Metadata.

Metadata	Description
Name	Name of the image
Dataset-Name	Dataset name
Owner	Ownership of the images if available
System	Acquisition system
Orientation	Specifify in which direction the images are acquired: Nadir, 45 or DHP^*a*^
Date	Date in format: mm-yyyy. Ex 06-2019
Species	Crop type
Latitude	Latitude in WGS84
Longitude	Latitude in WGS84
LocAcc	Location Accuracy = Boolean 0 (GPS location is approximate) or 1 (GPS location is accurate)
TVT-split{1–5}	Specify image category for Training/Validation/Test for the five different VegAnn splits

^*a*^DHP refer to images from Digital Hemispherical Photography.

**Table 3:** IOU and *F*_1_ score averaged over the five cross-validation fold of VegAnn computed at the dataset and image-level.

Model	Encoder	*IOUimage*	*F*_1_*image*	*IOUdataset*	*F*_1_*dataset*
Unet	Resnet34	86.0 ± 1.2	91.0 ± 0.9	89.7 ± 1.4	94.5 ± 0.8
Unet	Resnet50	86.3 ± 0.8	91.4 ± 0.8	90.0 ± 0.5	94.8 ± 0.3
DeepLabV3	Resnet34	85.4 ± 0.6	90.8 ± 0.5	89.5 ± 0.2	94.5 ± 0.2
DeepLabV3	Resnet50	84.9 ± 0.8	90.5 ± 0.7	89.0 ± 0.8	94.2 ± 0.5

**Table 4:** The IOU and *F*_1_ scores, averaged over the five test sets of VegAnn computed for images acquired using various acquisition systems available in VegAnn.

System	*IOUdataset*	*F*_1_*dataset*
Handeld Cameras	90.4 ± 1.1	95.0 ± 0.6
DHP	90.9 ± 2.1	95.2 ± 1.2
UAV	90.9	95.3
Phenomobile	94.6 ± 2.3	97.2 ± 1.2
Phone Camera	85.5 ± 1.0	92.2 ± 0.6

To achieve this objective, several steps were followed:

- The first criterion was to prioritize the diversity of locations and select as many locations as possible. Among series corresponding to the same acquisition conditions, e.g. same location and date, we selected a single image.
- We used a stratified random sampling to include images representing all the phenological stages of the crops.
- We carried out a second round of image selection by training a deep learning model on a subset of the first selection. A U-net, a fully convolutional neural network with a standard^30^ encoder-decoder architecture and ResNet34 backbone implemented in the^31^ library was used for this purpose. A visual inspection of the results allowed us to identify the type of images and domains (e.g crop type and stage, conditions of acquisition) that were not well represented and we could then include these in the final version of VegAnn.

Table 1 summarizes the characteristics of the datasets used to compose VegAnn which originates from two scientific communities, e.g. plant phenotyping and satellite remote sensing.

**Fig. 3:**
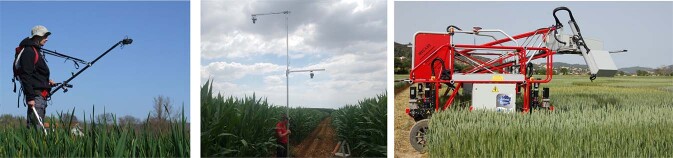
Three acquisition systems used to build VegAnn. From left to right: handheld camera on the LITERAL device, camera equipped with a fish-eye lens and mounted on a probe, and cameras mounted on the Phenomobile. The individuals consent to the publication of the images.

The LITERAL dataset was acquired with a handheld system called LITERAL (Fig. 3). An operator maintains a boom with a pair of Sony RX0 cameras fixed at its extremity. The 938 images covered a wide range of different cereal crop species grown at several places in France. Wheat images from the GWHD Global Wheat Head Detection^25,32^ from France and China (Nanjing) are also included in this dataset.

The PHENOMOBILE dataset was acquired with the Phenomobile system, an unmanned ground vehicle. This system uses flash lights synchronized with images image acquisition making the measurements independent from the natural illumination conditions.

INVITA (INnovations in Variety Testing in Australia) is a project led by The University of Queensland in Australia that aims to monitors the quality and performances of wheat variety trials^33^. This dataset is constituted with a wide range of wheat crop cultivars grown in >100 different locations with photos collected with smartphones.

Easypcc is a dataset from the University of Tokyo. It is constituted of rice and wheat time series images acquired with a fix sensor in the field. Less variability can be found in this dataset since images are acquired at the same location but with different lighting conditions settings.

The P2S2 dataset^11^ was initially acquired for the validation of green cover fraction products derived from decametric resolution satellite (e.g. SENTINEL-2). It is constituted of images of a spatial resolution of 0.2 mm. Nine crop species, four sites (in France and Belgium) and five measurements dates were monitored across the growing seasons.

The DHP dataset corresponds to patches extracted from digital hemispherical photographies (Fig. 7). The acquisition were performed to extract canopy structure characteristics from true-color image for the validation of Copernicus global land products derived from medium spatial resolution satellite observation^34^.

Thus, it covers various crops, locations, and growing scenarios and includes some shrubs, herbaceous wetlands, grasslands pasture and herbaceous.

The Crowdsourcing dataset was constituted with diverse crop images assembled from diverse sources included from the web. It is mostly images acquired with smartphones. A proportion of the images (41) correspond to bare soils (e.g. background pixels with no vegetation) and were collected to better represent the variability of soil backgrounds in VegAnn.

**Fig. 4:**
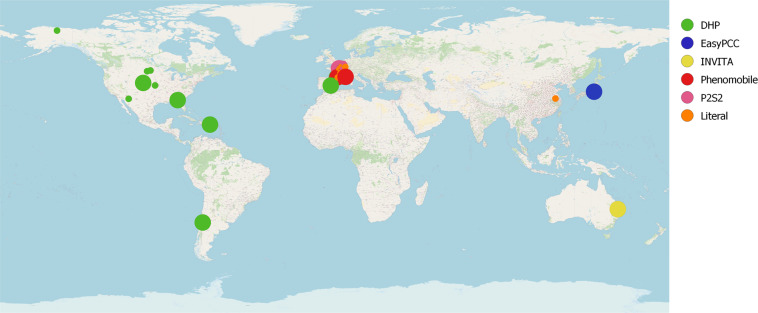
Location of the crop sites where VegAnn images were acquired. The color indicates the original dataset from which the raw images were selected and the radius of the points is proportional to the number of images acquired at each site.

**Fig. 5:**
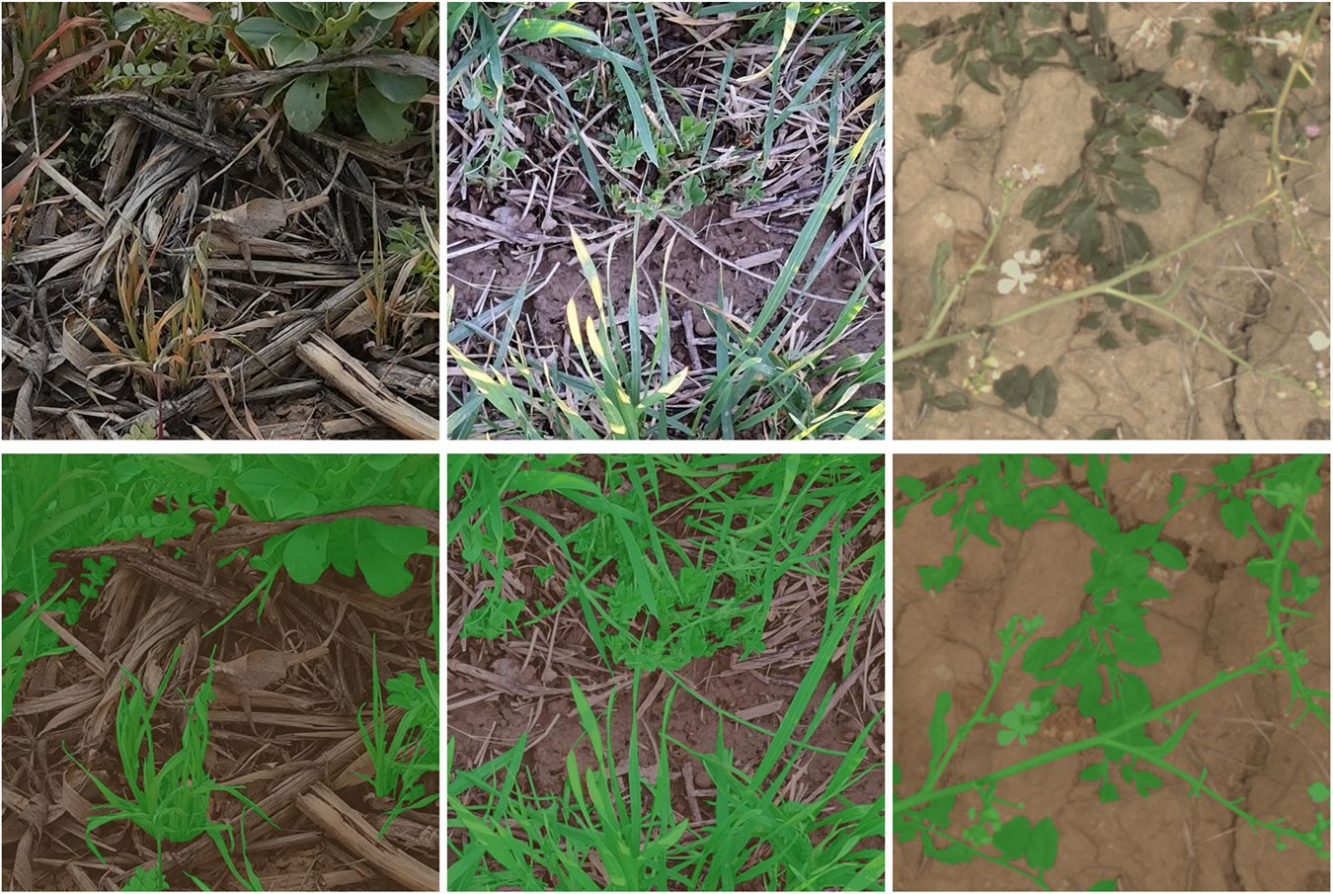
Raw images and their background/vegetation labels. From left to right: examples taken from Literal, Crowdsourcing and Phenomobile sub-datasets (Table 1). Images are from mixed crops cultivated in agroecology.

We refer the readers to the available references for more details about the different datasets. Figure 3 shows examples of the different acquisition platforms that are used to compose VegAnn. Figure 4 displays image location with respect to their datasets and number of images and Fig. 5 shows example of images along with their labels.

### VegAnn metadata and characteristics

In this section we describe the metadata, listed in Table 2, that are associated with each image contained in VegAnn.

#### Dataset Name

The DatasetName corresponds to the initial dataset from which the image was extracted (see Table 1)

#### Latitude, longitude and loccAcc

The GPS information in WGS84 coordinate reference system is stored in the Latitude and Longitude attributes. The attribute LocAcc is a boolean set to 1 if the location is exact and 0 if the location has been approximated due to missing information.

#### System of acquisition

**Fig. 6:**
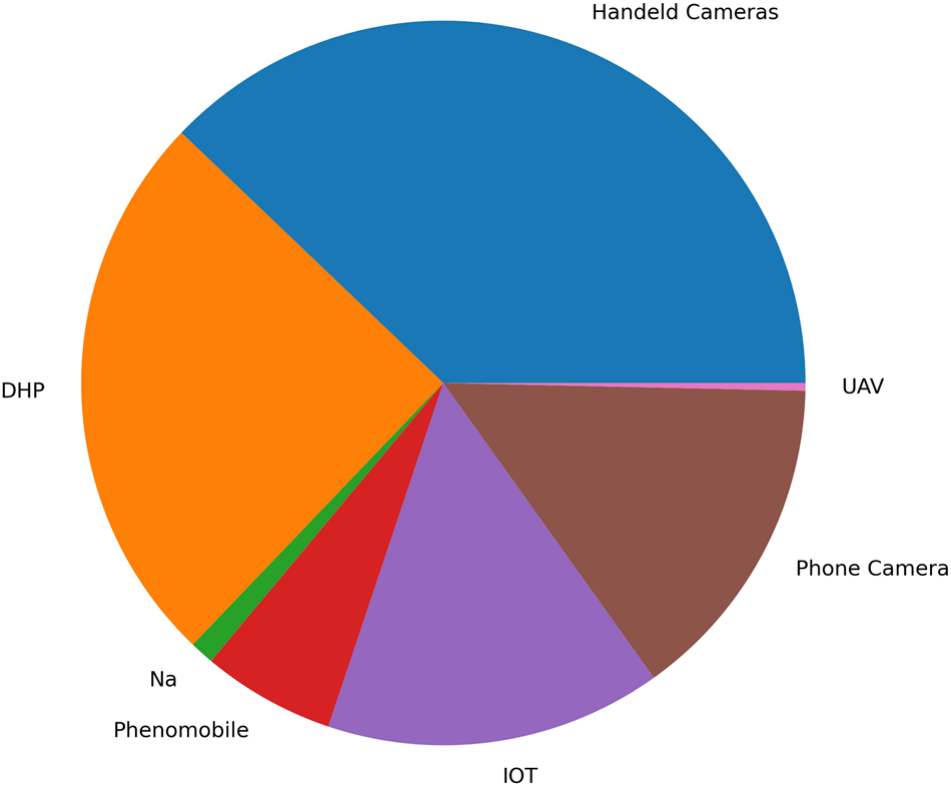
Proportion of VegAnn images acquired with the different systems.

**Fig. 7:**
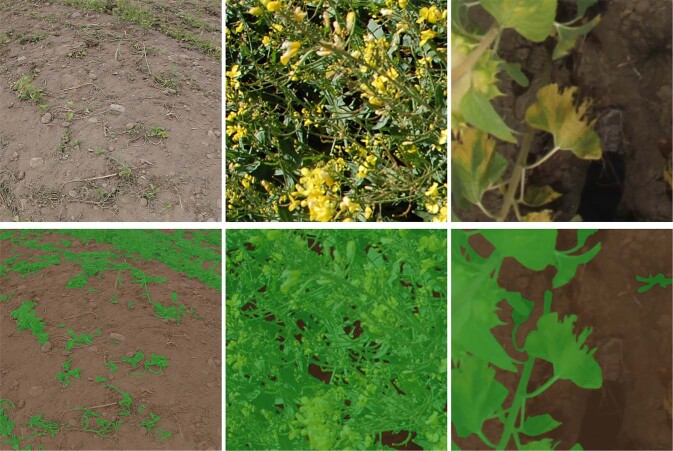
Example of DHP images and their corresponding vegetation/background labels.

Six different acquisition systems were used to build the VegAnn dataset and the corresponding proportion of images per system is shown in Fig. 6. **Handeld cameras** refers to high resolution commercial cameras, held by an operator with a boom or a tripod at 60–80 cm above the canopy (Fig. 3). **DHP** images were acquired by an operator using downward looking cameras equipped with a fish-eye lens, at around 60–80 cm above the canopy. Due to the field of view of fish-eye lens, the pixels of a DHP image represent quite different viewing orientations as compared to the **Handeld cameras** (Fig. 7). **IOT** refers to fixed camera placed in the field and looking downward, at height of 20–60 cm from the crop, depending on the growth stage. **Phone Camera** were acquired with conventional smartphones, and such images are generally associated with a lower quality. **Phenomobile** images were acquired with a mobile robot under controlled illumination conditions 3, by synchronising a flash with the acquisition. A few images were acquired with a camera mounted on unmanned aerial vehicles (**UAV**) flying at low altitude. Finally, it was not possible to determine the origin of a few images and are tagged as **Na** referring to unknown system of acquisition.

#### Orientation

Four different viewing information can be found in VegAnn: **nadir**: the viewing direction is close to the nadir (e.g vertical) with a small camera field of view; **45** the images were acquired with a camera inclined at 45° (Literal and Phenomobile datasets); **DHP** image extracted from hemispherical images, for which the viewing direction is unkonwn and very variable within the image due to the large field of view of the fish-eye lens. Finally, **Na** indicates that the viewing direction is unknown (crowdsourcing dataset).

#### Species

**Fig. 8:**
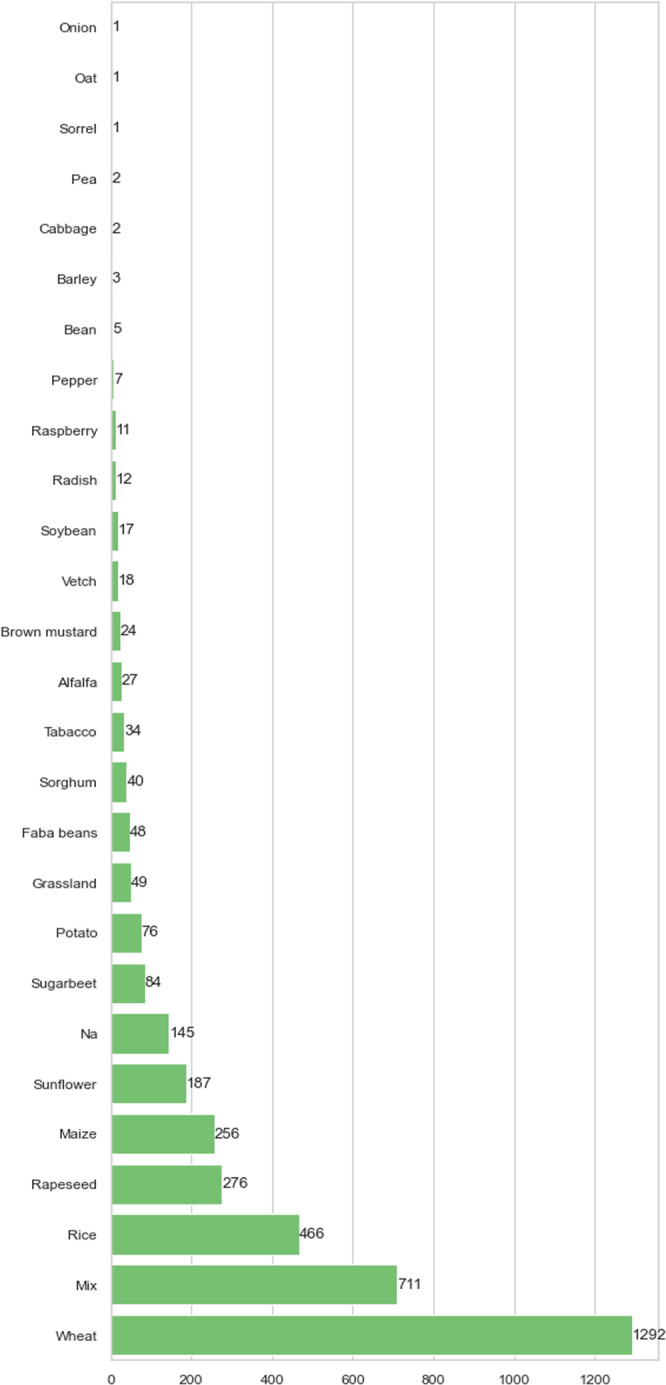
Crop type distribution in VegAnn.

The VegAnn dataset contains images from 26 crop types at different phenological stages, and grown under various pedo-climatic conditions (Fig. 8). A high proportion of crops characterized by small leaves have been included since small leaves combined with an irregular spacing and high overlap between plants make pixel wise segmentation of the vegetation more challenging. Therefore, wheat and rice are highly represented since they are the most widely cultivated and studied small leaf crops in the world. To complement the representativeness of this kind of canopy structure, we included a high proportion of more complex canopies composed of at least two species 4 (Mix: crops with weeds or mixed crops cultivated in agroecology). Images acquired over bigger leafed crops of various shapes and sizes were also selected to incorporate some of the most cultivated and studied crops in the world (potato, sugarbeet, sunflower and maize). However, they are in a lower proportion since their labelling is comparatively easier.

#### Training/Validation/Test sets of VegAnn

As VegAnn was primarily built for benchmarking segmentation approaches, we provide five distinct Training/Validation/Test (TVT) sets.

To generate these TVT sets, we randomly selected five crops that were represented by fewer than 100 images, namely Vetch, Brown Mustard, Potato, Sorghum, and Sugarbeet. In each TVT set, one of these five crops was included in the Test dataset, as follows: Set 1 (Vetch), Set 2 (Brown Mustard), Set 3 (Potato), Set 4 (Sorghum), and Set 5 (Sugarbeet).

In order to develop models that generalize across different domains, we ensure that images with the same species, acquisition date, and coordinates were not present in the same set, we created the training, validation, and test datasets separately. However, in some cases where too many images were available for the same species, acquisition date, and coordinates, we were unable to avoid such occurrences. Note that we included the images from the dataset EasyPCC acquired with a fix sensor in the field in the training sets. We aimed for a distribution of approximately 85%, 5%, and 15% in the training, validation, and test datasets, respectively, for each TVT set.

The attribute “TVT-split1” indicates the category to which the images belong in Set 1, “TVT-split2” for Set 2, and so on.

## Data Records

The dataset can be downloaded from Zenodo: 10.5281/zenodo.7636408^28^ and is under the CC-BY license, allowing for reuse without restrictions. Images are of 512 pixels × 512 pixels and are saved in 8-bit PNG format. Images and their associated labels are stored in the “images” and “annotations” folder with the same file name. Meta information can be found in the VegAnn-dataset.csv file and is described in the following sections. All the available attributes are listed in Table 2.

## Technical Validation

The labeling work was subcontracted to a private company that offers labeling services by Photoshop experts. Each labelled image was then carefully verified by at least two agronomy experts from our team and was re-annotated if required. The images without consensus (lack of illumination, poor quality, fuzzy) were eventually excluded from the dataset.

The technical validity of the VegAnn annotations was ensured by the iterative process used to construct the dataset. This was carried out in two ways:

1. During the labelling phase, independent visual review of the labels of each image, by at least two persons
2. While training and evaluating different deep learning approaches for automatic background/vegetation segmentation with VegAnn, the images leading to poor segmentation performances were carefully checked to understand whether these poor performances were due to the approach or to the labelling. When necessary, the labelling was corrected and reviewed once again.

There are different possible usages of VegAnn. Considering the uniqueness of VegAnn in terms of crop species, crop phenological stages, pedo-climatic conditions, and acquisition conditions, the main use would be the benchmarking and the updating of segmentation approaches for crops. Other usages could also be foreseen: as the raw images are labelled with a crop type, they could be used to complement other datasets for automatic crop recognition, or the validation of land use maps. As an illustration of the potential of VegAnn, we used this dataset to train and evaluate a deep learning model to segment vegetation from background in images acquired over crops. This work was further used to estimate the canopy structure (gap fraction, leaf area index, proportion of senescent vegetation) in phenotyping experiments^29^ and used for the automatic processing of the P2S2 hemispherical images to derive ground truth for the validation of satellite leaf area index products^9^.

### Evaluation scores

We used the 5 fold sets provided by VegAnn and computed baseline metrics to evaluate the performances of the approach. The Intersection Over Union (IOU) and *normalF*_1_ score of the pixel predictions at the dataset- and image-level over the five folds were computed. The results obtained over the five folds were then averaged and reported with their standard deviation. It should be noted that the metrics reported at the dataset-level are in fact aggregated over the whole dataset and do not correspond to metrics averaged over each image. We recommend users to refer to the metrics at the dataset-level and not at the image level to reduce the influence of “empty” images i.e. images without vegetation.

1 $$IOU=\frac{TP}{TP+FP+FN}\quad \quad Precision=\frac{TP}{TP+FP}\quad \quad Recall=\frac{TN}{TP+TN}\quad \quad {F}_{1}=2.\frac{Precision.Recall}{Precision+Recall}$$

### Implementation details

The models were implemented in PyTorch version 1.10 with PyTorch lightning framework. For this first evaluation we utilize fully convolutional neural network with a standard encoder-decoder architecture. Two variation: Unet^30^ and DeeplabV3^35^ were used. ResNet34 and ResNet50 backbones implemented in the^31^ library were used. The model weights were initialized on Imagenet^36^. We trained our models using Adam optimizer^37^, with a learning rate of $1e-4$ and Dice loss as the cost function. The batch size was fixed at 16 and the training process was conducted for 15 epochs. More detail about the implementation can be found in https://github.com/simonMadec/VegAnn.

### Evaluation of the dataset

We report the performances averaged over the 5 official cross-validation folds of VegAnn in Table 3.

Regarding the Unet model architecture and Resnet34 backbone feature extractor: an average IOU of 86.0% and 89.7%, at the image and dataset-level respectively, were achieved over the five folds of VegAnn. Although different models and encoders were tested, the results showed only marginal differences between them. These results of the binary vegetation/background classification might be deemed satisfactory and leave plenty of room for improvements. The different metrics remain quite stable over the five folds (standard deviation over the five folds at the dataset level is 1.4% for IOU and 0.8% for IOU), indicating the robustness of the approach.

**Fig. 9:**
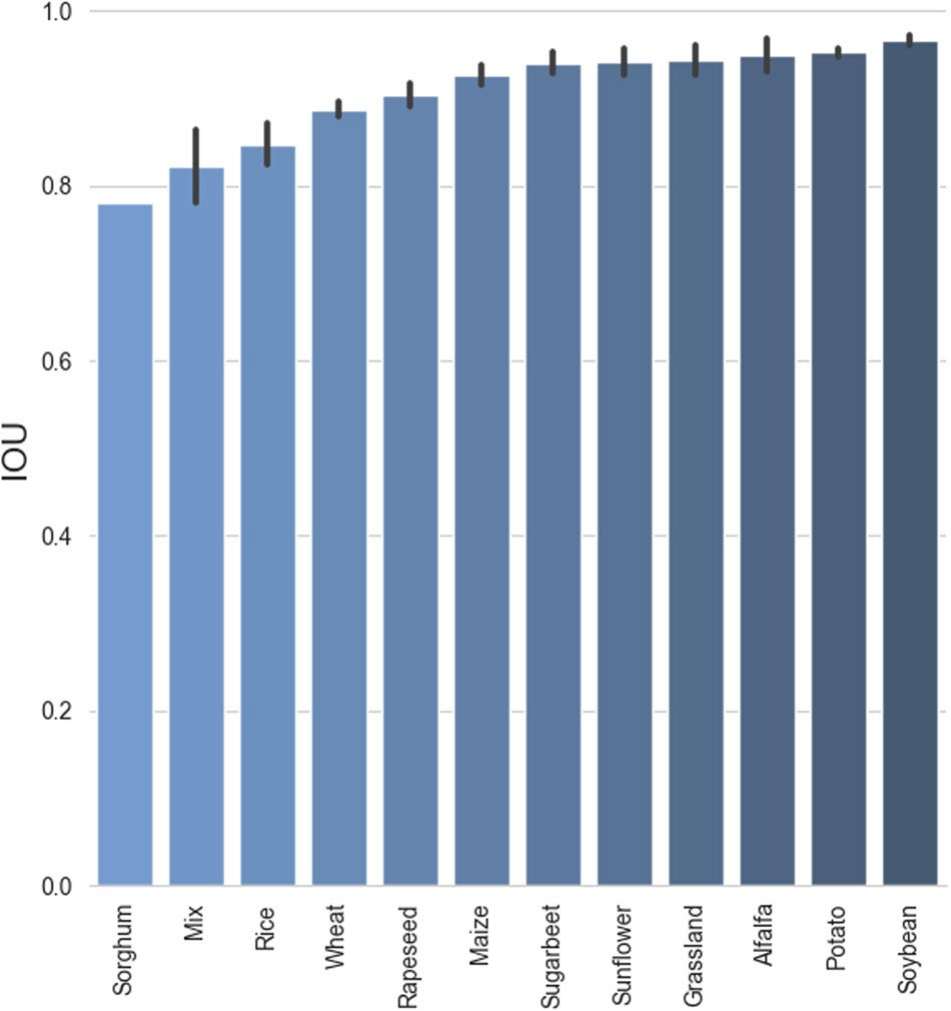
The performances of the segmentation model evaluated per species over the five test folds of VegAnn - the mean IOU along with the standard deviation in the form of error bars are reported.

**Fig. 10:**
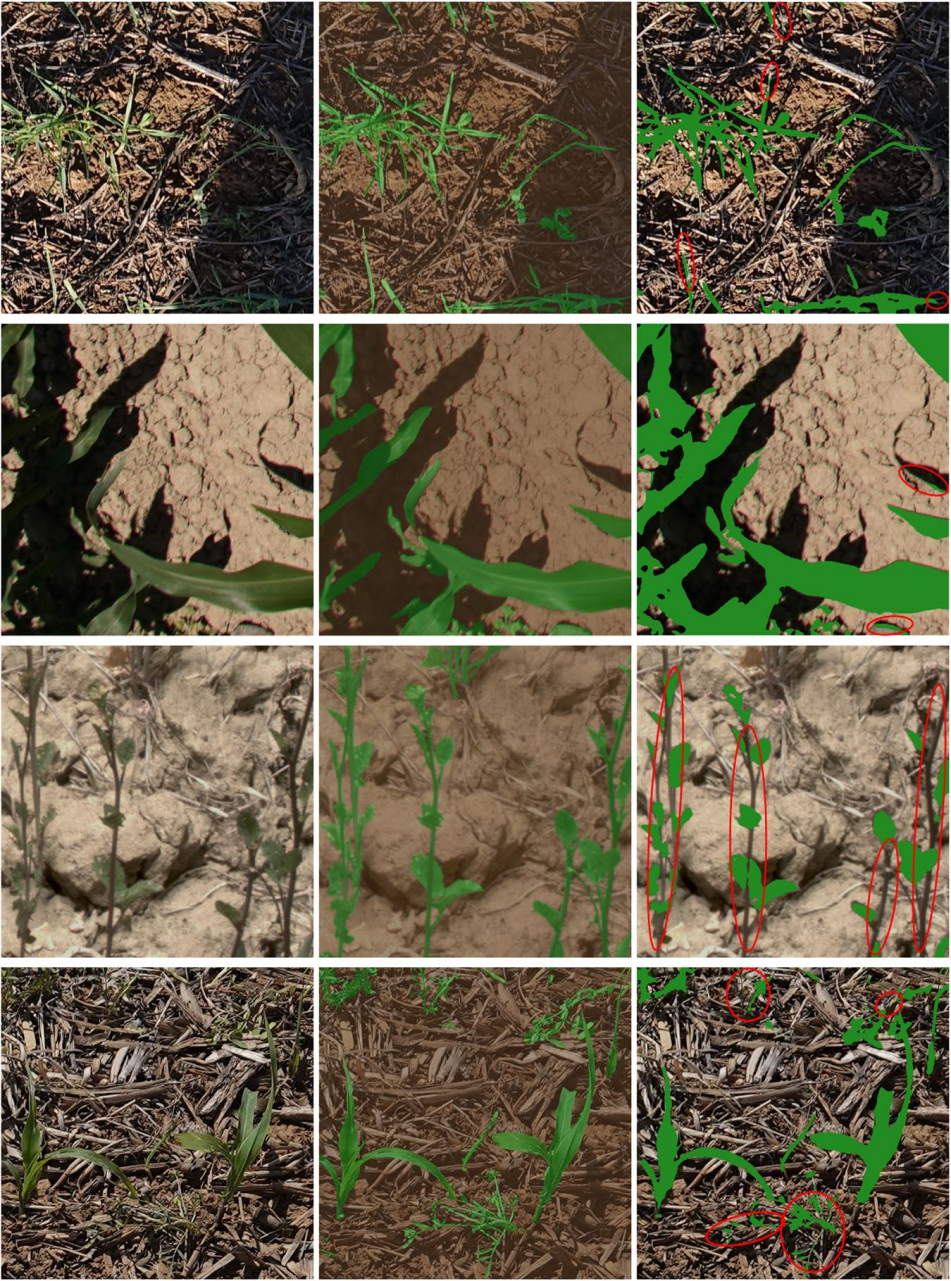
Visualization of segmentation results on VegAnn test set where the model faces some difficulties. The scene is quite complex and lacks texture and color information to confidently between vegetation and background.

The IOU scores computed over the different species present in the test folds of VegAnn are summarized in Fig. 9. Species with a low number of images may not be present in the test fold of VegAnn and are not reported in this figure. Several visualizations of the model predictions, along with the ground-truth masks are also presented in Fig. 10.

The baseline approach presented in this study faces challenges when classifying scenes acquired from certain species. As observed in Fig. 10, these difficulties may arise due to various reasons, such as poor image quality, scene complexity, configuration of the sensor or acquisition set-up. For instance, the Sorghum images obtained from the VegAnn dataset were acquired using unmanned aerial vehicles and DHP cameras, which led to a lower spatial resolution. The lower results reported for the Mix, Wheat, and Rapeseed categories could also be attributed to the complexity of these scenes 10.

Table 4 shows the per-system results using the VegAnn generic approach. The highest performance was achieved for images captured under controlled illumination conditions with the phenomobile robot, whereas images acquired with a smartphone had the lowest performance. However, other factors, including the crop types, could have influenced these results. However, other factors, including the crop types, could have influenced these results. Notably, the majority of images captured with phone cameras depicted wheat, which is a challenging crop to segment.

**Fig. 11:**
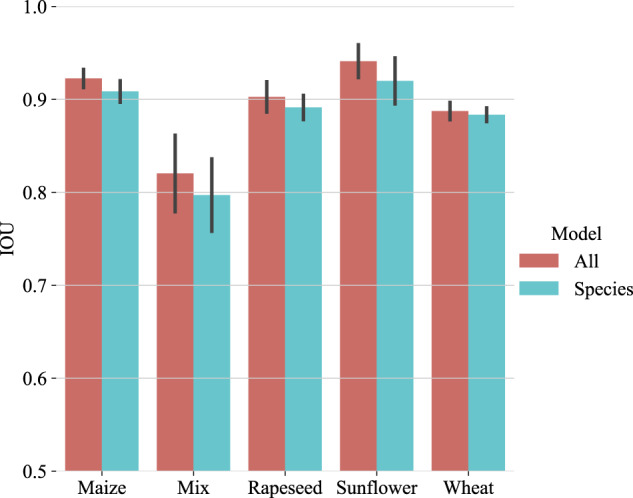
Comparing the performances of the segmentation models trained using the crop specific approach (species in blue) and the generic VegAnn approach (All i.e. all crop types in red) and evaluated over the VegAnn test datasets.

Additionally, we also compare a crop-specific learning approach i.e. a vegetation/background segmentation model trained on images acquired over a single crop, with the VegAnn generic approach i.e. a vegetation/background segmentation model trained on images acquired over all crop species. The comparisons were performed separately for each crop. For the crop-specific learning approach, we only considered crop species with a sufficiently large number of images in both the Training and Test sets, which included maize, rapeseed, mixed crop, sunflower and wheat (Fig. 8). The VegAnn generic approach provides better results than the crop-specific approach, with an average of the IOU of 1.5 point and lesser variability among the five folds for all the species. (Fig. 11). This illustrates the strength gained by merging images of different crops to improve background detection, as it strengthens the model by leveraging the diversity of the images.

## Data Availability

The codes to reproduce the baseline results presented in the Usage Notes section is available at https://github.com/simonMadec/VegAnn. We recommend users to start with th custom PyTorch dataloader to run easily for instance the training/evaluation with the crop-specific approach and the VegAnn generic approach, more information can be found in the associated ReadMe file.
